# Microevolution of antimicrobial resistance and biofilm formation of *Salmonella* Typhimurium during persistence on pig farms

**DOI:** 10.1038/s41598-019-45216-w

**Published:** 2019-06-20

**Authors:** Eleonora Tassinari, Geraldine Duffy, Matt Bawn, Catherine M. Burgess, Evonne M. McCabe, Peadar G. Lawlor, Gillian Gardiner, Robert A. Kingsley

**Affiliations:** 1 Quadram Institute Bioscience, Norwich Research Park, Norwich, UK; 2 Teagasc, Food Research Centre, Ashtown Dublin, 15 Ireland; 3 Earlham Institute, Norwich Research Park, Norwich, UK; 4 Teagasc Pig Development Department, Animal & Grassland Research & Innovation Centre, Moorepark, Fermoy, Co. Cork, Ireland; 5 Department of Science, Waterford Institute of Technology, Waterford, Ireland; 6 University of East Anglia, Norwich, UK

**Keywords:** Pathogens, Bacterial genetics

## Abstract

*Salmonella* Typhimurium and its monophasic variant *S*. 4,[5],12:i:- are the dominant serotypes associated with pigs in many countries. We investigated their population structure on nine farms using whole genome sequencing, and their genotypic and phenotypic variation. The population structure revealed the presence of phylogenetically distinct clades consisting of closely related clones of *S*. Typhimurium or *S*. 4,[5],12:i:- on each pig farm, that persisted between production cycles. All the *S*. 4,[5],12:i:- strains carried the *Salmonella* genomic island-4 (SGI-4), which confers resistance to heavy metals, and half of the strains contained the mTmV prophage, harbouring the *sopE* virulence gene. Most clonal groups were highly drug resistant due to the presence of multiple antimicrobial resistance (AMR) genes, and two clades exhibited evidence of recent on-farm plasmid-mediated acquisition of additional AMR genes, including an IncHI2 plasmid. Biofilm formation was highly variable but had a strong phylogenetic signature. Strains capable of forming biofilm with the greatest biomass were from the *S*. 4,[5],12:i:- and *S*. Typhimurium DT104 clades, the two dominant pandemic clones found over the last 25 years. On-farm microevolution resulted in enhanced biofilm formation in subsequent production cycle.

## Introduction

*Salmonella* is the second most common cause of food borne disease in many countries world-wide and is associated with more deaths than any other foodborne disease in resource rich countries^1,2^. Pork and pork products are a leading source of human salmonellosis in the European Union^3,4^. The risk of pig meat contamination is exacerbated by the high prevalence of *Salmonella* in pigs arriving at slaughter, often in the absence of overt signs of disease^5–8^. Furthermore, antimicrobial resistance of *Salmonella* in pigs is a global concern, with resistance to at least one antimicrobial observed in 92% of *Salmonella* isolates in the UK^9^. Consequently, a reduction in the prevalence of *Salmonella* in pigs arriving at slaughter is a high priority for many countries, and subject to national control programmes^7^. Efforts to control *Salmonella* on farms is by a combination of endeavour to use *Salmonella*-free feed, good biosecurity measures, appropriate antibiotic usage, and implementation of an all-in all-out production system allowing for cleaning and disinfection between production cycles^7,10^. Although cleaning and disinfection of pig housing between production cycles is effective at reducing residual contamination, it is not completely effective as *Salmonella* may persist on water drinkers, feeders, and floor and wall surfaces, particularly if these are damaged^7^. The mechanisms used by *Salmonella* to persist in the farm environment are not known, but the ability to form biofilms is thought to be an important factor impacting survival in the environment^11^. The continued widespread use of antibiotics to treat or prevent disease has resulted in a high level of antibiotic resistance in *Salmonella* isolated from pigs, impacting the effectiveness of treatment on farm and dissemination into the food chain^12,13^.

Historically, *Salmonella enterica* serotype Typhimurium (*S*. Typhimurium), defined by the antigenic formula 4,[5],12:i:1,2, is the serotype most frequently isolated on pig farms^9,14–17^. Since around 2005 the frequency of isolation of a monophasic variant of *S*. Typhimurium (*S*. 4,[5],12:i:-) has increased in many parts of the world^18–24^. The epidemiological record of *S*. Typhimurium in livestock in England and Wales, and in Germany between 1970 and 2010 was characterized by a succession of dominant multidrug resistant (MDR) clones, namely DT204, DT104 and the current *S*. 4,[5],12:i:- DT193/DT120 strains^25,26^. Initially, DT204 and DT104 emerged in cattle, but spread to pigs and poultry^26,27^. In contrast, *S*. 4,[5],12:i:- DT193/DT120 strains emerged initially in pigs and have only recently spread to cattle and poultry in the UK where they remain minority types^19,27,28^. In Ireland, a study of *Salmonella* sampled from manure on pig farms in 2009–2010 reported *S*. Typhimurium DT104 as the predominant type^17^ and *S*. 4,[5],12:i:- was first reported in pig herds in 2012, suggesting a rapid clonal replacement^29^. A second MDR strain of *S*. Typhimurium phage type U288 emerged in UK pigs around 2001 accounting for up to 50% of all isolates but is rarely isolated from other livestock and poultry^27,30^.

A ubiquitous trait of historic and currently dominant strains of *S*. Typhimurium is MDR. *S*. Typhimurium DT104 exhibits a resistance profile of ACSSuT (ampicillin, chloramphenicol, streptomycin, sulphonamides, tetracycline) encoded within a complex type I integron on *Salmonella* genomic island 1 (SGI-1), an integrative mobilisable element^31^. *S*. Typhimurium U288 strains typically exhibit a resistance profile of ACSSuTTm (including trimethoprim) due to two independent insertions into a pSLT plasmid^32^. *S*. 4,[5],12:i:- DT193/DT120 strains exhibit an ASSuT resistance profile encoded on two resistance regions (RR1 and RR2) that are flanked by IS26 sequence and present adjacent to one another near the *fljB* locus that encodes the phase II flagellin protein^33^. Insertion of RR1 and RR2 appears to have resulted in multiple deletions in the *fljB* locus and adjacent sequence that results in the monophasic phenotype of *S*. 4,[5],12:i:-, but no apparent impact on pathogenicity^34,35^. Although an MDR phenotype appears to be key to the success of epidemic strains, it does not explain the succession of dominant strains. A characteristic of the *S*. 4,[5],12:i:- DT193/DT120 epidemic strains is the presence of both *cus* and *pco* genes involved in copper and silver homeostasis present on an 80 kb genetic island, termed SGI-4^34,36^. The *cus* and *pco* genes may impart an advantage for circulation in pig herds where pharmacological levels of copper are added to feed due to its non-specific antimicrobial properties^37^. Further genotypic variation within the epidemic clade is generated by the acquisition, on multiple occasions, of a novel prophage (mTmV), encoding the SopE type III secretion system effector protein^34^, that contributes to host cell invasion and induction of pro-inflammatory diarrhoea^38–40^.

To investigate persistence on pig farms and potential transmission from feed mills we determined the phylogenetic relationship of 138 *S*. 4,[5],12:i:- and *S*. Typhimurium strains isolated from pig farms in Ireland on two occasions crossing production cycles using whole genome sequencing^41^. We then addressed the hypothesis that persistence on pig farms was associated with microevolution affecting drug resistance or biofilm formation, characteristics potentially selected during production or cleaning between production cycles. The distribution of AMR genes on mobile genetic elements was determined in order to study patterns of acquisition. The ability to form biofilms was investigated in the context of the phylogeny to investigate farm-specific phenotypes and to determine its association (if any) with persistence.

## Materials and Methods

### Bacterial strains and culture conditions

Isolation of *Salmonella* on ten pig farms located across three provinces of the Republic of Ireland on two sampling occasions in successive production cycles was described previously^41^. Briefly, ten farrow-to-finish pig farms (farms A-J) with a history of high *Salmonella* prevalence (>50%) were sampled twice between March 2012 and June 2013. Samples were collected across all production stages and from faeces, feed, water and the farm environment including floors, walls, water drinkers and troughs. *S*. Typhimurium or *S*. 4,[5],12:i:- was isolated from all farms except farm E. Therefore, isolates from nine farms were included in this study. Isolation from feed from three commercial feed mills (mills B, C and D) and one home compounder (mill E) were described previously^29^. We retain farm and mill designations for ease of reference. For routine culture, strains were grown in Luria Bertani Broth with shaking at 37 °C. For determination of red, dry and rough (RDAR) phenotype, strains were cultured on Congo Red agar plates for 48 hours at 28 °C. For conjugation experiments, donor and recipient strains were cultured for 18 hours in 5 ml of LB broth at 37 °C without shaking. Cells were harvested by centrifugation, re-suspended in phosphate buffered saline pH7 and donor and recipient strains were mixed in a 1:1 ratio in 5 ml LB broth to OD_600nm_ of 0.1 and incubated without shaking for 24 hours at 26 °C. Ex-conjugants were enumerated on LB agar supplemented with nalidixic acid (50 µg/ml) or nalidixic acid (50 µg/ml)/chloramphenicol (25 µg/ml) and incubated at 30 °C for 18 hours, and CFU enumerated. Transfer of pSTM6-275 to the recipient strain was determined by PCR using primers 5′-TTTCTCCTGAGTCACCTGTTAACAC-3′ and 5′-GGCTCACTACCGTTGTCATCCT-3′, that annealed specifically to IncHI2 plasmids replicon. MIC for antibiotics was performed using a microdilution assay, as previously described^34^.

### Biofilm assay

The formation of biofilm on polystyrene was assessed using a microtiter plate assay as previously described^42^. Bacterial strains were cultured overnight in 5 ml LB broth without salt at 37 °C under static conditions. The OD_600nm_ of the culture was subsequently adjusted to 0.02 using LB broth without salt, and 200 µl aliquots were dispensed in eight wells (8 technical replicates per biological replicate) of a microtiter plate. The 96-well plates were then incubated at 22 °C to simulate the environmental temperature of pig farms, without shaking, for 24 or 48 hours, after which the supernatant was removed, and the cells washed with 200 μl of tryptone salt (Oxoid Limited), before fixing with 300 µl of pure ethanol for 20 minutes. Biomass was stained with crystal violet and the OD was read at 595 nm. At least two biological replicates were performed.

### Genomic DNA preparation and sequencing

The genomic DNA of 104 *S*. 4,[5],12:i:- and 34 *S*. Typhimurium strains was isolated using a Wizard® Genomic DNA purification kit (Promega). Sequencing libraries were prepared using the Low Input Transposase-Enabled (LITE) library developed at the Earlham institute (EI) and 125 bp paired-end reads were generated using Illumina HiSeq 2500 with version 4 chemistry and in High Output mode according to manufacturer’s instructions (Illumina).

### DNA sequence analysis and phylogenetic reconstruction

For phylogenetic reconstruction, single nucleotide polymorphisms (SNPs) were identified in the whole genome sequences of 138 *S*. 4,[5],12:i:- and *S*. Typhimurium strains with reference to *S*. Typhimurium strain SL1344 by aligning sequence using BWA MEM^43^, variant calling with Freebayes^44^ and SNP filtering using vcflib/vcftools^45^, using Snippy v1.0^46^. Maximum-likelihood trees were constructed using a general time reversible (GTR) substitution model with gamma correction for among-site rate variation with RAxML v8.0.20^47^. Rapid bootstrapping was performed with 450 replicates. Suspected recombined sequence was removed to improve the accuracy of the phylogeny using Gubbins v2.3.4^48^. The size of the reference genome in which variant sites were used for phylogenetic reconstruction changed depending on the dataset used. For 138 isolates mapped to strain SL1344, variant and invariant sites alignment was 3,824,106 bases long, corresponding to the 78.4% of the reference genome, for the subset of these used for analysis of variation in biofilm formation this was 2,328 informative SNPs in 4,213,370 bases (86%) of the SL1344 reference genome. The phylogeny of the IncHI2 plasmid was based on 162 variant sites within the 193,845 base-long core-genome (70% of reference plasmid).

Targeted assembly of IncHI2 plasmid sequence was performed using a bespoke pipeline; sequence reads were mapped against *S*. Typhimurium L01157-10 assembled sequence using Snippy^46^ and the unmapped reads were then isolated and assembled using VelvetOptimiser^49^. The contigs generated were then merged and their ability to circularise tested with Circlator^50^. The homology of the IncHI2 plasmids with the pSTM6-275 plasmid (accession number CP019647.1) was visualised using the BRIG^51^.

The presence of sequence reads mapping to the *sopE*, SGI-4 and *fljAB* loci was investigated using SRST2 v0.1.7^52^ with custom databases. Matches with >90% coverage and <10% sequence divergence were reported as present. I*n silico* detection of AMR genes, integrons and plasmids from ResFinder, INTEGRALL, and PlasmidFinder databases was performed using ARIBA^53–57^ and visualised using ggtree^58^.

## Results

### Farm-specific genotypes of *S*. Typhimurium and *S*. 4,[5],12:i:- in Ireland can persist across production cycles

**Figure 1:**
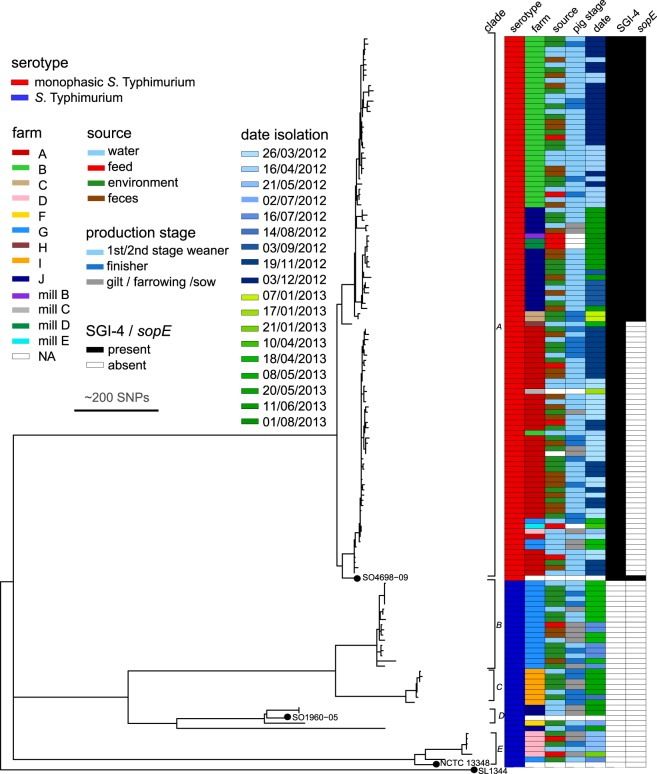
Maximum likelihood phylogenetic tree of isolates of *S*. Typhimurium and *S*. 4,[5],12:i:- from pig farms or feed mills in Ireland. The tree was constructed using 2,427 informative SNPs in the core genome of 138 isolates from nine farms or four feed mills with reference to *S*. Typhimurium strain SL1344 (accession number NC_016810.1). *S*. Typhimurium strains SL1344, strain NCTC13348 (phage type DT104) and strain SO1960-05 (phage type U288) were included in the tree for reference. The serotype, farm, source of isolation and stage of pig production, date and the presence of *Salmonella* genetic island-4 (SGI-4) and the *sopE* gene are indicated by filled boxes as indicated in the embedded key.

To establish the phylogenetic relationship of *S*. Typhimurium on nine pig farms and four feed mills in Ireland, we determined the whole genome sequence of 34 *S*. Typhimurium and 104 *S*. 4,[5],12:i:- (Table 1) from a previous study^41^. A maximum likelihood phylogenetic tree constructed using 2,427 informative SNPs from the core genome of the farm isolates and three reference strains SO4698-09 (*S*. 4,[5],12:i:- DT193/120), NCTC13348 (DT104) and SO1960-05 (U288) indicated the farm and feed isolates were present in five major clades (clades A to E, Fig. 1). In each case, clades consisted of strains distinguishable from a hypothetical common ancestor of the clade by 5–30 SNPs in the core genome. The majority of strains (104) were in clade A and were closely related to strain SO4698-09, a reference strain for the current MDR *S*. 4,[5],12:i:- which is part of a current pandemic^34^. Clade A contained at least one strain from seven of the nine farms. Clade E contained six strains from two farms and these were closely related to *S*. Typhimurium DT104 (NCTC13348, a reference strain for a previously dominant MDR pandemic clone)^59,60^. Three strains clustered with a reference strain for phage type U288 (strain SO1960-05), a representative from an ongoing epidemic of *S*. Typhimurium in pigs in the UK^61^. The remaining strains formed two distinct but closely related subclades (B and C). We observed a strong phylogenetic signature for strains from each farm, with the majority of strains isolated from each farm closely related to one another. *S*. Typhimurium and *S*. 4,[5],12:i:- were isolated from a wide range of sources on most farms, including the environment within the pens and troughs, water drinkers, feed and in the freshly voided faeces (Fig. 1). In many cases, identical or very closely related strains (<5 SNPs in core genome) were isolated from multiple pens, troughs, feeders and pigs, on the same farm suggesting their spread on the farm.

**Table 1:** Farm, production stage, source of isolation and antimicrobial resistance profile of the *S*. 4,[5],12:i:- and *S*. Typhimurium strains used in the study, from Burns *et al*. 2015 and Burns *et al.* 2018.

Serotype (n)	Farm/feed mills	Production stage^a^	Pig Faecal isolates (%)	Environmental isolates^b^ (%)	Feed isolates (%)	Water isolates (%)	Antimicrobial resistance profile (%)
*S*. 4,[5],12:i:-(n = 104)	A, B, C, D, G, H, J, mill B, mill C, mill D, mill E	FW, G, W1, W2, F	28 (26.9%)	54 (52%)	10 (9.6%)	12 (11.5%)	ASSuTTm (33.7%) ASSuT (30.8%) ACSSuTTmGm (8.7%) Others (26.8%)
*S*. Typhimurium (n = 34)	D, F, G, I, J	FW, G, W1, W2, D, F	3 (8.8%)	22 (64.7%)	3 (8.8%)	6 (17.7%)	Susceptible (32.4%) T (20.6%) ASSuT (8.8%) ACSSuT (5.8%) ACSSuTTm (5.8%) Others (26.6%)

^a^FW: farrowing; G: gilts: W1: 1^st^ stage weaner; W2: 2^nd^ stage weaner; D: dry sow; F: finisher. ^b^Environmental samples from swabs from the pen, water drinkers, feed troughs and feed bins.

In four cases, strains isolated from two different farms or a farm and feed mill had identical or fewer than 5 SNPs in the core genome and therefore likely represented transmission events (Fig. 1). The core genome sequence of one *S*. 4,[5],12:i:- strain from farm B was identical to that of a number of strains from farm A. Indistinguishable strains from three different farms were identical to one another, and to an isolate recovered from soybean meal sampled from the home compounder mill E. Two strains from feed mill D were identical in core genome sequence to a strain isolated from farm J, and different by a single SNP from an isolate from mill B. Also, a single isolate from mill C was identical to two strains from farm A.

On six of the nine farms, *Salmonella* Typhimurium or *S*. 4,[5],12:i:- strains were isolated on two separate occasions, 8 to 9 months apart, from successive productions cycles (Fig. 1). In each case most strains isolated on the second sampling were closely related to the previously isolated strains, and the tree topology was consistent with direct descent from earlier strains, suggesting that these strains either persisted on the farm between production cycles or were reintroduced from a common source.

### Diverse antimicrobial resistance genes are associated with farm-specific strains

Multidrug resistance is common in *S*. Typhimurium, particularly strains isolated from farm animals in which antibiotic use is widespread^62^. We therefore analysed the whole genome sequence of strains to determine variation in AMR genes. Furthermore, since these genes are commonly encoded on plasmids we also identified replicon sequences (Supplementary Fig. 1).

**Figure 2:**
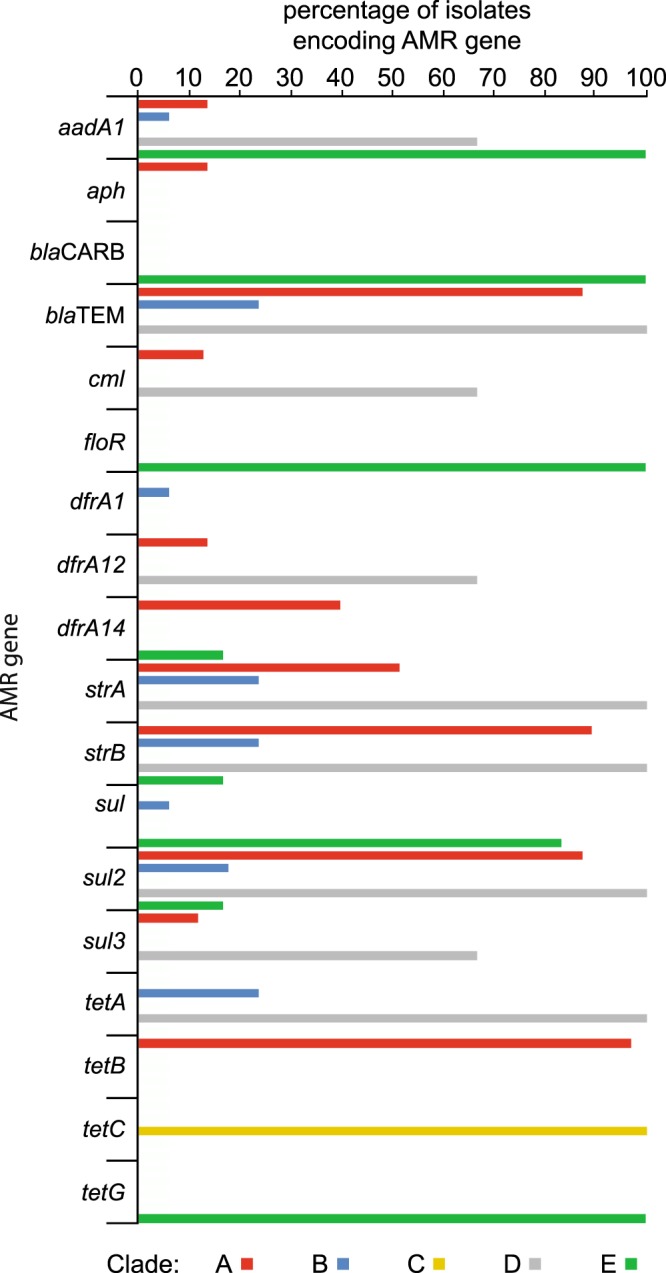
Distribution of antimicrobial resistance genes in the *S*. Typhimurium phylogenetic clades. Bars indicate the percentage of the isolates that encode each resistance gene. Clades correspond to those in Fig. 1. Clade A: *S*. 4,[5],12:i:- (n = 104), Clade B: *S*. Typhimurium (n = 17); clade C: *S*. Typhimurium (n = 7); clade D: *S*. Typhimurium U288 (n = 3); clade E: *S*. Typhimurium DT104 (n = 6). Genes identified were *aadA1/aadA2*: aminoglycosides resistance gene; *aph*: aminoglycosides resistance gene; *blaCARB*: carbenicillin resistance; *blaTEM*: penicillins/early cephalosporins resistance; *cmI*: chloramphenicol resistance; *floR*: florfenicol/chloramphenicol resistance gene; *dfrA1*/*dfrA12*/*dfrA14*: trimethoprim resistance; *strA*/*strB*: streptomycin resistance genes; *sul1/sul2/sul3*: sulphonamide resistance genes; *tetA*/*tetB*/*tetC*/*tetG*: tetracycline resistance; *mefA/mefB*: macrolide, lincosamide and streptogramin B resistance; *msrD*: macrolide resistance; *qacEDelta1/qacH*: quaternary ammonium compounds.

Generally, the presence of AMR genes correlated with phenotypic resistance in laboratory tests with few exceptions (Supplementary Fig. 1). Only 13 strains lacked resistance genes entirely. The distribution of AMR genes differentiated phylogenetic clades, but considerable variation occurred within clades containing strains that differed by 5–10 SNPs, indicating rapid and ongoing gene flux on farms (Fig. 2). Strains within the epidemic clade of *S*. 4,[5],12:i:- and *S*. Typhimurium DT104 encode MDR on the chromosome, located on mobile genetic elements^33,34,63,64^, and *S*. Typhimurium U288 in integrons and transposons on a pSLT-like plasmid^61^. Six *S*. Typhimurium strains that were closely related to the previous epidemic DT104 type strain (NCTC13348), encoded genes consistent with the common ACSSuT penta-resistance profile of this clone, as expected. They also encoded the *qacE* (Delta 1) gene, conferring resistance to quaternary ammonium compounds, previously described^65^. Most *S*. 4,[5],12:i:- contained the genes associated with the typical ASSuT tetra-resistance profile associated with the current epidemic clone^34^. The presence of the *bla*_TEM_, strA, *strB*, *sul2* and *tetA* genes in *S*. 4,[5],12:i:- strains correlated with an IncQ replicon sequence (*repA*) present in the RR1 and RR2 region of the chromosome^33^. However, 14 *S*. 4,[5],12:i:- had lost various combinations of the *bla*_TEM_, *strA*, *strB sul2* and *tetA* genes, likely due to mobilisation through various IS26 elements within RR1 and RR2 regions^33,34^. In addition, a large proportion of the strains carried *dfrA14* gene (trimethoprim) which may have inserted into *strA*, as suggested by its concomitant absence as described in several plasmids^66^. Four strains closely related to a representative *S*. Typhimurium U288 strain (clade D), were resistant to ampicillin (*bla*_*TEM-1b*_), streptomycin (*strA*, *strB*), sulphonamides (*sul2*, *sul3*) and tetracycline (*tetA* or *tetB*) consistent with previous descriptions of a U288 strain^32^. However, additional resistance genes to aminoglycosides (*aadA1/aadA2* and/or *aph*), chloramphenicol (*cmI*), trimethoprim (*dfrA12*), macrolides (*mefB*) and quaternary ammonium compounds (*qacH*) previously unreported in this clonal group were present in some strains.

### Acquisition of plasmids enhanced antimicrobial resistance on two farms

**Figure 3:**
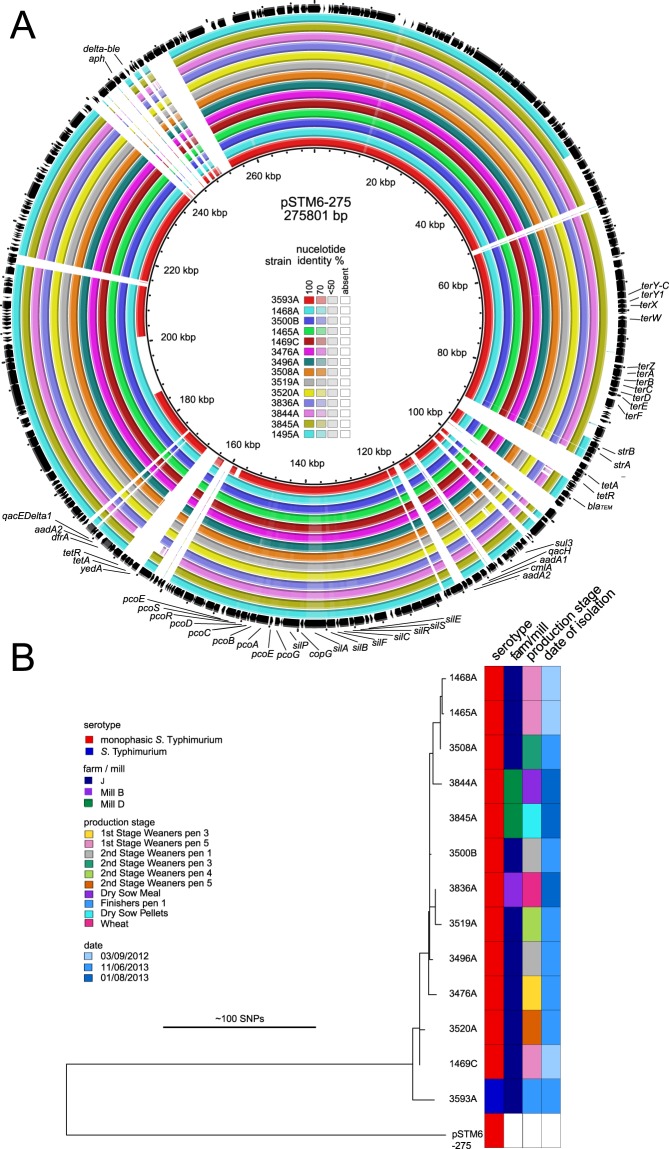
Sequence similarity of IncHI2 plasmids from Irish farms with the pSTM6-275 plasmid. (**A**) The extent of the homology between the Irish IncHI2 plasmids with the pSTM6-275 was visualised with BRIG. Sequence assembly contigs from each sample were blasted against the pSTM6-275, which was used as reference. Each circle represents the plasmid from each of the 14 Irish isolates. The colour gradient indicates the degree of nucleotide identity. Genes in pSTM6-275 are represented by arrows (outer circle). Genes encoding antimicrobial or heavy metals resistance genes are annotated. A detailed list of gene presence/absence is present in Supplementary Table 4. (**B**) Phylogenetic relatedness of IncHI2 plasmids. Maximum-likelihood phylogeny of the thirteen IncHI2 plasmid sequences based on 162 core-genome SNPs identified using pSTM6-275 as reference. Due to the high proportion of missing data, plasmid from isolate 1495 C was excluded from the phylogenetic analysis.

Two clusters of strains in clade A (*S*. 4,[5],12:i:-) and clade B encoded additional AMR genes that correlated with the presence of plasmid replicons IncHI2 and IncI, respectively (Supplementary Fig. 1). In each case strains containing the additional replicon were direct descendants of a common ancestor that was sensitive to these antibiotics and lacked the replicon, and in the case of clade B there was evidence from the tree structure for this having occurred on the farm. In the first example, 13 *S*. 4,[5],12:i:- strains encoded additional resistance genes, *aadA1/aadA2*, *aphA*, *cml*, *dfrA12, mefB, sul3*, and *qacH* conferring resistance to aminoglycosides, chloramphenicol, trimethoprim, macrolides, sulphonamides, and quaternary ammonium compounds. Ten of these strains were from farm J and three strains from two feed mills. All of the strains with the IncHI2 replicon were closely related, with the exception a single *S*. Typhimurium strain from farm J. IncHI2 plasmids have previously been reported in epidemic *S*. 4,[5],12:i:- strains in China and Australia, including pSTM6-275^67,68^. Comparison of assembled whole genome sequences with that of pSTM6-275 indicated a high level of sequence identity to 90% of the plasmid (Fig. 3A), with a number of short indels (Supplementary Table 4). Phylogenetic reconstruction using 162 core SNPs of the plasmid with reference to pSTM6-275 indicated that all of the plasmids from farm J and feed mills strains were closely related, including the *S*. Typhimurium strain (Fig. 3B), and relatively distantly related to pSTM6-275. The IncHI2 plasmid from *S*. Typhimurium strain 3593A was close to the root of the plasmid clade associated with farm J and two of the mills, suggesting a recent common source. Moreover, the plasmid from this isolate carried the *qacEDelta1* gene, which was absent from the plasmids from *S*. 4,[5],12:i:-. *S*. 4,[5],12:i:- strain 3508 A was able to transfer resistance to aminoglycosides, chloramphenicol, trimethoprim and sulphonamides by conjugation *in vitro* with a frequency of 4 × 10^−3^ per recipient. Although the plasmid could not be visualised by horizontal gel electrophoresis, as described for large plasmids previously^69^, sequence was detected by PCR amplification using specific oligonucleotide primers. Also, the presence of *mefB* in U288-like isolates (Clade D) from farm J and in IncHI2-positive isolates may suggest its plasmid-independent mobilisation between distinct genotypes on this farm.

An example of probable on-farm acquisition of a plasmid encoding AMR genes was observed on farm G. Sixteen *S*. Typhimurium strains from this farm formed a phylogenetically distinct clonal group (clade B, Fig. 1), 13 of which were pan-susceptible and four acquired *bla*_*TEM*-1b_, *strA*, *strB*, *sul2* and *tetA* AMR genes that correlated with the presence of an IncI1 replicon (Supplementary Fig. 1). All three MDR strains were present on distal branches of the phylogenetic tree and were isolated during a second sample collection on the farm, suggesting that a strain that persisted over production cycles on the farm had acquired the IncI1 plasmid.

### SGI-4, *fljB* locus and *sopE* gene flux in *S*. 4,[5],12:i:- farm strains

We previously reported the microevolution and genotypic variation of *S*. 4,[5],12:i:- strains in the UK and Italy^34^. *S*. 4,[5],12:i:- strains were distinct from all other *Salmonella* reported previously in encoding an 80 kb genetic island termed SGI-4, adjacent to the yjdC and Phe-tRNA loci, that encodes genes conferring enhanced resistance to copper and potentially other metals, such as silver and arsenic. Three of 78 strains investigated previously lacked SGI-4, due to deletion^34^. All of the *S*. 4,[5],12:i:- strains in the current study encoded SGI-4, further supporting the potential significance of this island in the success of the clone (Fig. 1). A second reported source of genotypic variation was the acquisition of a novel phage termed mTmV, that carried the *sopE* gene^38,39^. Approximately half of the *S*. 4,[5],12:i:- strains contained the *sopE* gene, with strains from three farms and two feed mills containing the gene (Fig. 1). Further genotypic variation resulted from multiple deletions in and adjacent to the *fljB* locus (Supplementary Fig. 2). Deletion of the *fljB* locus was associated with the lack of the second phase flagella antigen, but deletions varied in size as previously reported, affecting from 3 to 25 genes. Finally, the absence of IncFI plasmids within the epidemic monophasic variant is noteworthy.

### *S*. Typhimurium and *S*. 4,[5],12:i:- farm isolates exhibit diverse expression of cellulose, curli fimbriae and the ability to form biofilm

**Figure 4:**
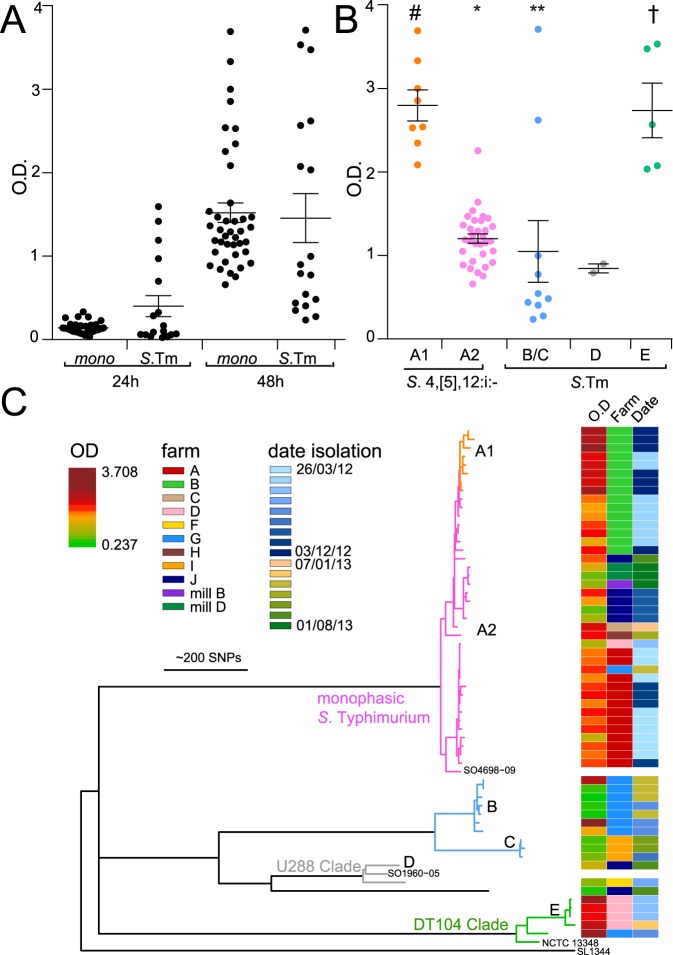
Biofilm formation by *S*. Typhimurium and *S*. 4,[5]12:i:- strains and correlation with the phylogeny. The biofilm-forming ability of the isolates was investigated with the microtiter plate assay. (**A**) Scatterplot of the OD_595nm_ values measured for *S*. 4,[5],12:i:- and *S*. Typhimurium after 24 and 48 hours of incubation at 22 °C. (**B**) Comparison of strains from each clade (A1, A2, B–E) to form biofilm. The OD_595nm_ values measured after 48 hours of incubation were plotted based on the topology of the phylogenetic tree. Pairwise Mann-Whitney test (95% of confidence interval) was performed and p < 0.05 were indicated as; p < 0.05 for all except clade E isolates (DT104-like) (#),: p < 0.05 for all clades (**),: p < 0.05 for all except clade A1 (†). (**C**) Maximum-likelihood phylogenetic tree as Fig. 1 with a subset of isolates for which biofilm formation was determined. The heatmap shows OD_595nm_ measured after incubation at 22 °C for 48 hours. The clade designation is indicated.

The ability to form a biofilm is thought to increase survival in the environment by increasing resistance to desiccation, sheer forces, and antimicrobial compounds such as biocides and antibiotics^11^. Cellulose and curli fimbriae constitute two of the key components forming the extra-cellular matrix (ECM) which covers *Salmonella* biofilms and production correlates with the thickness of the biofilms^70^. The majority of both *S*. 4,[5],12:i:- (98.1% %) and *S*. Typhimurium (76.7%) strains exhibited a red, dry and rough (RDAR) phenotype on Congo red agar after 72 hours at 28 degrees, indicating production of cellulose and curli fimbriae. The remaining strains produced a pale and smooth phenotype. The ability of farm strains to form biofilm on a polystyrene surface in 96-well plates was similar for *S*. Typhimurium and *S*. 4,[5],12:i:- strains at 22 °C for 24 and 48 hours incubation (Fig. 4A). Both *S*. Typhimurium and *S*. 4,[5],12:i:- formed a significantly greater biofilm after 48 hours of incubation at 22 °C, compared to 24 hours (p < 0.005, Fig. 4A) and biofilm formation at 37 °C was low for both *S*. Typhimurium and *S*. 4,[5],12:i:- strains (data not shown).

Despite the mean biofilm formation being similar for both *S*. Typhimurium and *S*. 4,[5],12:i:- strains, considerable variability in biofilm formation was observed after 48 hours incubation at 22 °C (Fig. 4A). In general, there was a high degree of congruence of the amount of biomass in biofilm and the phylogenetic relatedness of strains (Fig. 4B,C). *S*. Typhimurium exhibited a greater variation in biofilm formation than *S*. 4,[5],12:i:- strains. Most of the *S*. Typhimurium strains that formed strong biofilms were closely related to the DT104 reference strain (Clade E). The remaining *S*. Typhimurium strains in three separate clades (Clades B, C and D) generally formed moderate or weak biofilms, with the exception of two strains that formed strong biofilms. Despite the fact that all the *S*. 4,[5],12:i:- strains were very closely related, biofilm formation appeared bimodal, with a large number of strains forming moderate biofilm levels (clade A2) and eight strains accumulating significantly more biomass (clade A1). All nine *S*. 4,[5],12:i:- strains that exhibited the greatest level of biofilm formation were isolated from farm B, and were distinct from all other *S*. 4,[5],12:i:- strains by virtue of the presence of a non-synonymous substitution present in the *edd* gene, encoding a phosphogluconate dehydratase that participates in the Entner-Doudoroff glycolytic pathway.

## Discussion

*S*. Typhimurium and the monophasic variant *S*. 4,[5],12:i:- are the most commonly isolated serotype from pigs, especially in finisher herds where intestinal carriage at slaughter is a primary risk factor for contamination of the food chain^8,71^. An understanding of the colonisation, persistence and transmission of *S*. Typhimurium on farms, and variation in genotype and phenotype is therefore key to the rational design of interventions aimed at amelioration of the risk to food safety. Whole genome sequencing provided the resolution to distinguish virtually all strains, and where no differences in sequence were detected, the confidence that these were the same strain isolated on two separate occasions. Furthermore, in this study phylogenetic reconstruction based on sequence variation in the core genome was used to infer ancestry, and to interpret gene flux associated with AMR on the farm.

The phylogenetic relationship of strains from farms in Ireland suggested that each had been colonised in the recent past by a clone of *S*. Typhimurium or *S*. 4,[5],12:i:- and undergone limited sequence divergence. Most strains isolated from each farm differed by 0–12 SNPs in the core genome and were more closely related to one another, than to strains isolated from other farms. However, in some cases identical or near identical strains were isolated from more than one farm or feed mill, particularly within the *S*. 4,[5],12:i:- clade. For example, a subclade of *S*. 4,[5],12:i:- formed from 41 strains from farm A, also contained a strain from each of farms B and D, and three strains from farm G. This pattern suggested either contamination of multiple farms from a common source, or direct transmission between the farms. The latter cannot be excluded as information regarding movement of animals between farms was not collected, but introduction through a common source is more likely, based on the implication of feed as an important source of *Salmonella* on farms^41^. That *S*. 4,[5],12:i:- strains from three commercial feed mills, one home feed compounder and at least some of the strains from seven of the nine farms were essentially clonal indicated a close relationship and recent transmission. In particular, the clonality of a group of strains from farm A with that from mill C, which supplied the farm, is consistent with at least initial transmission from contaminated feed to the farm at some point in the recent past.

The strong farm-specific phylogenetic signature was striking and the maintenance of this structure over two sampling occasions spanning 6–9 months suggested that *Salmonella* was most likely persistent on these farms. Furthermore, there was a moderate level of sequence divergence of strains isolated on each farm since they shared a common ancestor, suggesting that they may have been present on the farm for several years. *S*. 4,[5],12:i:- on farm J exhibited the greatest sequence divergence with many strains having accumulated 10–15 SNPs since sharing a common ancestor. Based on the reported short-term substitution rate of 1–2 SNPs per genome per year for *Salmonella* Typhimurium^60,72,73^, the common ancestor for farm-specific clusters existed in the last 2–7 years. This is also consistent with previous studies that used subgenomic typing methods^15,74,75^.

The population structure of *S*. Typhimurium and *S*. 4,[5],12:i:- strains was consistent with considerable transmission on each farm both within pens (between pigs of similar age) and between pens housing multiple production stages. *Salmonella* differing by 0–5 SNPs were isolated from multiple pens housing pigs at various stages of weaning, fattening, or breeding from freshly voided faeces, the environment within a pen, that included the floor, walls and feed trough, feed, and drinking water and drinking nipple. While we cannot exclude multiple and continuous acquisitions of *Salmonella* from the same feed source, the relatively low incidence of *Salmonella* contamination in feed reported previously^29^ would suggest that this is more likely to be transmission within the farm due to ineffective biosecurity.

Consistent with previous reports, antimicrobial resistance genes were common in *S*. Typhimurium and *S*. 4,[5],12:i:-^76^. Most *S*. Typhimurium DT104 and *S*. 4,[5],12:i:- DT193/DT120 strains contained genes consistent with the widely reported resistance profile^33,77^, with occasional loss of one or more of the genes as previously reported^34,64^. However, there were also two clear cases of acquisition of additional AMR genes on plasmids; within a clade of 4,[5],12:i:- strains on farm J, and within a *S*. Typhimurium clade on farm G (clade B, Fig. 1). The first case involved acquisition of a large plasmid with extensive sequence similarity to pSTM6-275, an IncHI2 plasmid encoding heavy metal resistance and AMR genes in *S*. 4,[5],12:i:-, reported previously in Australia^67,68^. The pSTM6-275 plasmid was present in 11 strains that had been isolated on two separate occasions from farm J, and three *S*. 4,[5],12:i:- strains isolated from two different feed mills. All of the strains containing pSTM6-275 were *S*. 4,[5],12:i:-, except for a single farm isolate of *S*. Typhimurium. Strains of *S*. 4,[5],12:i:- with or without the plasmid were present in samples from both 2012 and 2013, suggesting plasmid gain or loss was occurring on the farm. Feed is a possible source of the pSTM6-275-like plasmid, as it was present in strains from two feed mills which were almost identical to plasmid-containing strains on farm J. In a second example, we observed evidence of AMR gene flux mediated by an IncI1 plasmid on farm G. While all the strains from the first sampling date lacked the IncI1 plasmid, a large proportion almost identical strains from the same clade carried the plasmid nine months later. These observations are consistent with the acquisition of the plasmid on the farm, from an unknown source.

The mechanisms by which *Salmonella* persist on farms is not known, but the ability to form biofilms is thought to be key to survival in the environment^78^. Our analysis suggested that distinct clones of *S*. Typhimurium and *S*. 4,[5],12:i:- strains were isolated from farms spanning at least two production cycles which strongly suggests that they persisted in the environment despite the cleaning and disinfection that is normal practice for pig facilities between batches of pigs via a combination of mechanical means and chemical disinfection^10,42^. The benefit of enhanced cleaning and disinfection compared to standard procedures was recently demonstrated^10,42^. Furthermore, the clonal structure of *Salmonella* on each farm and observation of farm-specific clones suggest that persistence on the farm is the most likely reason for *Salmonella* presence on farms and not reintroduction onto the farm from an external source. We observed considerable variability in the ability of strains to form biofilms on polystyrene, with a particularly large degree of variation in biofilm formation in *S*. Typhimurium strains. Strains from clades B, C and D (U288) generally exhibiting limited biofilm formation while clade E (DT104) exhibited the greatest biofilm formation (Fig. 4). *S*. 4,[5],12:i:- strains generally formed moderate to strong biofilms, and one sub-clade composed of strains from a single farm exhibited a significantly greater biomass in their biofilm compared to other *S*. 4,[5],12:i:- strains, similar to previous observations^42^. Most of the *S*. 4,[5],12:- strains circulating in Portugal could produce a strong biofilm and the ability of the strains to form biofilms increased between 2006 and 2011, suggesting that this provided an advantage for persistence^79^. Similarly, closely related *S*. 4,[5],12:i:- strains on farm B differed in biofilm formation with the second sampling time point exhibited significantly greater biofilm formation than those isolated earlier. The former group of strains differed from the latter by a single nucleotide polymorphism that resulted in an amino acid substitution in the *edd* gene, encoding an enzyme in the Entner-Doudoroff (ED) metabolic pathway^80^, that was previously implicated in biofilm formation of *Campylobacter jejuni*^81^. However, the ability to form a biofilm was not essential for persistence on the farm, since strains of clade B generally formed weak biofilms but were isolated from successive production stages on farm G.

Together these data highlight the remarkable clonality of *S*. Typhimurium or *S*. 4,[5],12:i:- on individual pig farms. However, gene flux frequency was high enough that even within highly clonal groups changes in coding capacity has the potential to affect virulence, flagella antigen expression, and antimicrobial and heavy metal resistance genes. Furthermore, variation in ability to form biofilms was dependent on the genotype of *S*. Typhimurium or *S*. 4,[5],12:i:-, supported by a strong phylogenetic signature for this phenotype, suggesting that intervention strategies aimed at decreasing the incidence of *Salmonella* on pig farms may vary in effectiveness depending on the prevalent genotype.

## Supplementary information

Supplementary Table 1

Supplementary Table 2

Supplementary Table 3

Supplementary Table 4

Supplementary Information
