# Supplementary material for: Microevolution of antimicrobial resistance and biofilm formation of Salmonella Typhimurium during persistence on pig farms
Source: Sci Rep. 2019 Jun 20;9:8832. doi: 10.1038/s41598-019-45216-w (PMC6586642; doi:10.1038/s41598-019-45216-w)
Supplement: Supplementary file 5 — Supplementary Information [file 41598_2019_45216_MOESM5_ESM.docx]

**Microevolution of antimicrobial resistance and biofilm formation of *Salmonella* Typhimurium during persistence on pig farms**

Running title: Genomic epidemiology of *Salmonella* on farms

Eleonora Tassinari^1,2^, Geraldine Duffy^2^*, Matt Bawn^1,3^, Catherine M. Burgess^2^, Evonne M. McCabe ^2^, Peadar G. Lawlor^4^**,** Gillian Gardiner^5^, Robert A. Kingsley^1,6^*

^1^Quadram Institute Bioscience, Norwich Research Park, Norwich, UK

^2^Teagasc, Food Research Centre, Ashtown, Dublin 15, Ireland

^3^Earlham Institute, Norwich Research Park, Norwich, UK

^4^Teagasc Pig Development Department, Animal & Grassland Research & Innovation Centre, Moorepark, Fermoy, Co. Cork, Ireland

^5^Department of Science, Waterford Institute of Technology, Waterford, Ireland

^6^University of East Anglia, Norwich, UK

**Supplementary Table 1. *S*. Typhimurium and *S*. 4,[5],12:i:- strains used in the study and relevant information.**

**Supplementary Table 2. Informative SNPs sites used to generate the maximum-likelihood phylogenetic tree of 138 *S*. 4,[5],12:i:- and *S*. Typhimurium in Figures 1, supplementary Figure 1 and Supplementary Figure 2.**

**Supplementary Table 3. Table with informative SNPs sites used to generate the maximum-likelihood phylogenetic tree of 58 *S*. 4,[5],12:i:- and *S*. Typhimurium showed in figures 4.**

**Supplementary Table 4. Gene presence/absence in IncHI2 plasmid with reference to plasmid pSTM6-275.**

**Supplementary Figure 1. Presence of antimicrobial resistance genes and plasmids in the *S*. 4,[5],12:i:- and *S*. Typhimurium isolates.**

The presence of antimicrobial resistance genes (red) and plasmids (green) is shown in the context of the maximum-likelihood tree described in figure 1. The antimicrobial resistance determinants and the plasmid replicons were identified from the ResFinder^56^, INTEGRALL ^57^ and PlasmidFinder^55^ databases, respectively, using ARIBA^54^. The serotype, farm, and the date of isolation are reported as well.

**Supplementary Figure 2. Deletion of the *fljAB* locus and flanking sequence.** The deletion of the *fljAB* locus and surrounding ORFs is shown within the phylogenetic context (maximum-likelihood phylogenetic tree described in figure 1). The gene presence/absence was assessed with srst2^52^ using the representative *S*. Typhimurium strain LT2 (accession number AE006468.2) as reference, Minimum gene coverage: 90%.
